# Selective Neuromuscular Denervation in Taiwanese Severe SMA Mouse Can Be Reversed by Morpholino Antisense Oligonucleotides

**DOI:** 10.1371/journal.pone.0154723

**Published:** 2016-04-28

**Authors:** Te-Lin Lin, Tai-Heng Chen, Ya-Yun Hsu, Yu-Hua Cheng, Bi-Tzen Juang, Yuh-Jyh Jong

**Affiliations:** 1 Graduate Institute of Medicine, College of Medicine, Kaohsiung Medical University, Kaohsiung, Taiwan; 2 Division of Pediatric Emergency, Department of Emergency, Kaohsiung Medical University and Kaohsiung Medical University Hospital, Kaohsiung, Taiwan; 3 Department of Biological Science and Technology, College of Biological Science and Technology, National Chiao Tung University, Hsinchu, Taiwan; 4 Graduate Institute of Clinical Medicine, College of Medicine, Kaohsiung Medical University, Kaohsiung, Taiwan; 5 Institute of Molecular Medicine and Bioengineering, College of Biological Science and Technology, National Chiao Tung University, Hsinchu, Taiwan; 6 Departments of Pediatrics and Laboratory Medicine, Kaohsiung Medical University Hospital, Kaohsiung Medical University, Kaohsiung, Taiwan; University of Edinburgh, UNITED KINGDOM

## Abstract

Spinal muscular atrophy (SMA) is an autosomal recessive motor neuron disease caused by deficiency of the survival of motor neuron (SMN) protein, which leads to synaptic defects and spinal motor neuron death. Neuromuscular junction (NMJ) abnormalities have been found to be involved in SMA pathogenesis in the SMNΔ7 SMA mouse model. However, whether similar NMJ pathological findings present in another commonly used mouse model, the Taiwanese SMA mouse, has not been fully investigated. To examine the NMJs of the Taiwanese severe SMA mouse model (*Smn*^*-/-*^; *SMN2*^*tg/0*^), which is characterized by severe phenotype and death before postnatal day (P) 9, we investigated 25 axial and appendicular muscles from P1 to P9. We labelled the muscles with anti-neurofilament and anti-synaptophysin antibodies for nerve terminals and α-bungarotoxin for acetylcholine receptors (AChRs). We found that severe NMJ denervation (<50% fully innervated endplates) selectively occurred in the flexor digitorum brevis 2 and 3 (FDB-2/3) muscles from P5, and an increased percentage of fully denervated endplates correlated with SMA progression. Furthermore, synaptophysin signals were absent at the endplate compared to control littermate mice, suggesting that vesicle transport might only be affected at the end stage. Subsequently, we treated the Taiwanese severe SMA mice with morpholino (MO) antisense oligonucleotides (80 μg/g) via subcutaneous injection at P0. We found that MO significantly reversed the NMJ denervation in FDB-2/3 muscles and extended the survival of Taiwanese severe SMA mice. We conclude that early NMJ denervation in the FDB-2/3 muscles of Taiwanese severe SMA mice can be reversed by MO treatment. The FDB-2/3 muscles of Taiwanese severe SMA mice provide a very sensitive platform for assessing the effectiveness of drug treatments in SMA preclinical studies.

## Introduction

Spinal muscular atrophy (SMA) is an autosomal recessive motor neuron disease characterized by α- motor neuron loss in the anterior horn of the spinal cord [1] accompanied by muscle atrophy and weakness [2]. SMA, a leading genetic cause of hereditary infant mortality [3], is caused by deletion or mutation of the survival of motor neuron 1 (*SMN1*) gene [4]. The copy gene of *SMN1*, called *SMN2*, differs at a single nucleotide in exon 7 (C to T), resulting in exon 7 skipping during SMN mRNA transcription [5]. Therefore, the skipped exon 7 transcript (Δ7) can only be translated into a partially functional and truncated SMN protein. In general, *SMN1* and *SMN2* genes encode 90% and 10% of the full-length SMN protein, respectively. Clinically, SMA patients have functional loss of the *SMN1* gene, but they retain at least one copy of the *SMN2* gene [6]. There is still no effective treatment for SMA. Therefore, the creation of a suitable animal model of SMA is a crucial step for therapeutic discovery. In 2000, Taiwanese researchers introduced human *SMN2* to mice, which rescued the embryonic lethality and resulted in mice with different phenotypes resembling human SMA [7]. Accordingly, these SMA mice are classified into three phenotypes: severe form SMA mice, who die before postnatal day (P) 9; mice with intermediate severity, who die at approximately 2–4 weeks; and mild form SMA mice, who survive and breed normally [7].

Abnormal neuromuscular junctions (NMJs) have been observed in the SMA mouse models, including neurofilament accumulation at nerve terminals [8, 9], immature endplates [10–12], and reduced transmitter release [9, 13–15]. Previous studies on Δ7 mice indicated that the NMJs of the hind limb muscles were still fully innervated, even at the end stage [9, 16]. Moreover, recent studies have demonstrated that axial and appendicular muscles, including the serratus posterior inferior (SPI), masseter, longissimus capitis, and flexor digitorum brevis (FDB) muscles show severe NMJ denervation (< 50% fully innervated endplates) at the end stage (P14) of Δ7 mice [17]. Another recent study suggested that the NMJs of the lumbrical muscles of the hind-paw were vulnerable in Δ7 mice, as well as in amyotrophic lateral sclerosis (ALS) SOD1^G93A^ mice [18]. NMJ pathology in Δ7 mice has been regarded as a sensitive platform to evaluate the efficiency of potential therapeutics in SMA [19–22]. Correspondingly, recent studies have also shown neuromuscular transmission failures and smaller endplate size in Taiwanese mild SMA mice [23] and neurofilament accumulation in Taiwanese severe SMA mice [24, 25]. Taiwanese severe SMA mice have a more rapid disease progression and relatively simpler genetic background than Δ7 mice [7, 23], and therefore, may more suitably serve as an alternative platform for SMA drugs screening. Indeed, Taiwanese SMA mice have been widely used in the discovery of various drugs [25–31]. However, the NMJ phenotypes in the Taiwanese severe SMA mice are not well characterized, in contrast to the Δ7 mice. In the present study, we extensively examined the NMJs of the axial and appendicular muscles of Taiwanese severe SMA mice. We found that distal limb muscles, including the FDB-2 and FDB-3 muscles of Taiwanese severe SMA mice were most severely NMJ denervated and can be reversed by early morpholino (MO) antisense oligonucleotides (ASOs) treatment. These results imply that Taiwanese severe SMA mice may serve as a suitable platform for clarifying potential mechanisms driving selective NMJ denervation and evaluating preclinical drug efficacy for alleviating NMJ defects.

## Materials and Methods

### Animal Model

All animals used for this study and all protocols involving the use of animals were approved by Institutional Animal Care and Use Committee of Kaohsiung Medical University (approval ID: 98189, 101145). The Taiwanese SMA (*Smn*^-/-^; *SMN2*) mice were generated as previously described [7]. Mild SMA mice carry homozygous *SMN2* transgenes (*Smn*^-/-^; *SMN*2^tg/tg^). These mice develop necrotic ears and tails and live longer than 1 year. To generate severe SMA mice, the *Smn*^-/-^; *SMN2*^tg/tg^ mice were crossbred to heterozygous *Smn* knockout mice (*Smn*^+/-^; *SMN*2^0/0^) to generate 50% severe SMA mice (*Smn*^-/-^; *SMN*2^tg/0^) and 50% control littermates (control) (*Smn*^+/-^; *SMN*2^tg/0^). We confirmed two *SMN2* copies in severe SMA mice (*Smn*^-/-^; *SMN*2^tg/0^) and four *SMN2* copies in mild mice (*Smn*^-/-^; *SMN*2^tg/tg^) by capillary electrophoresis as described previously [32]. The *SMN2* and *Smn* knockout alleles were genotyped by PCR analyses of tail DNA [31]. Tail snips were collected at P0, and mice were identified by paw mark. The lifespan of Taiwanese severe SMA mice was about 9 days. Animals were allowed food and water ad libitum and were kept under constant temperature and controlled illumination conditions (lights on between 07:30 and 19:30). We checked the health of the mice at least twice a day, usually in the morning and afternoon. We used humane treatment for SMA mice when they showed paralysis of the hindlimb, weight loss, and poor appetite in the end stage. The mice were sacrificed by increased carbon dioxide, and decapitation was used for neonatal mice prior to P14 as an additional physical method to ensure death. There was no unexpected death during the study.

### Fixation and Dissection of Mouse Muscles

Mice of the desired genotype and age were anaesthetized by intraperitoneal injection of pentobarbital (5 mg/kg). After anaesthesia, we removed the right atrium and perfused 3 ml (depending on the weight of mice) phosphate-buffered saline (PBS) into the left ventricle, followed by 3 ml (depending on weight of mice) 4% paraformaldehyde. Mouse samples were stored in 4% paraformaldehyde at 4°C for 24 hours and transferred to PBS. We dissected 11 axial muscles, including the semispinalis capitis, latissimus dorsi, sternohyoid, masseter, diagastric posterior, trapezius, sternocleidomastoid, splenius capitis, intercostalis, longissimus capitus, and SPI, and 14 appendicular muscles, including the psoas, triceps brachii, quadriceps, gracilis, gluteus maximus, extensor digitorum longus (EDL), soleus, deltoid, gastrocnemius, anterior tibialis (AT), biceps brachii, and FDB. The FDB is anatomically divided into three slender muscles (FDB-2, FDB-3, and FDB-4), which flex the second, third, and fourth digits, respectively, upon contraction [33]. The dissection protocol was modified from that of a previous report [17].

### Immunohistochemistry of Neuromuscular Junctions

Whole muscles were teased into layers of 5–10 fibres in thickness for improved staining. The muscles were labelled with the following antibodies: chicken anti-neurofilament M (1:2000; Millipore) for neurofilament, and rabbit anti-synaptophysin (1:200, Invitrogen) for presynaptic nerve terminals. Acetylcholine receptors (AChRs) were labelled by Alexa Fluor 555-conjugated α-bungarotoxin (BTX) (1:200; Invitrogen). The secondary antibodies were Alexa Fluor 647 goat anti-chicken IgG (1:200; Invitrogen) and Alexa Fluor 488 goat anti-rabbit IgG (1:200; Invitrogen). All antibodies were mixed with 5% bovine serum albumin (BSA), 0.5% triton X-100, and 0.1% sodium azide and washed with washing solution: 0.5% triton X-100 in PBS. After staining, muscles were mounted with Fluor Preserve reagent (Calbiochem) on slides and stored at 4°C [17].

### Quantification of NMJ Morphology and Imaging

For the imaging of NMJs, all images were taken by confocal microscope (Zeiss LSM 740). Z-stack images of whole-mount muscles were obtained at sequential focal planes. Illustrated images were flattened projections of Z-stack images. For quantification of synaptic pathology, the NMJ was evaluated by categorizing endplates as fully innervated (neurofilament and synaptophysin signal labelling of more than 80% of the endplate), partially innervated (neurofilament and synaptophysin signal labelling of up to 80% of the endplate), and fully denervated (no neurofilament or synaptophysin overlying the endplate). For all analyses, 3 pairs of Taiwanese severe SMA and control mice were counted. For each muscle sample, one hundred NMJs were evaluated from a randomly selected field of the whole mount. Muscles with poor staining were excluded from analysis. To achieve unbiased analyses, observers were blinded to the genotypes of muscles [17]. For quantification of endplate size, all endplate sizes were estimated by manual tracing in Image J software to calculate the area. All analyses were performed on en-face endplates only, as well as on Z-stacks throughout the NMJs.

### Morpholino (MO) Antisense Oligonucleotide Treatment

The MO ASO sequence, numbered from the *SMN2* exon 7 donor site, was ATTCACTTTCATAATGCTGG (MWT = 6,754, Gene Tools) [34]. MOs were suspended in sterile 0.9% normal saline. Stock solutions were stored at -20°C, and working solutions were stored at 4°C. Four groups were treated by subcutaneous (SC) injection; severe SMA mice (*Smn*^*-/-*^*; SMN2*^*+/-*^) and control mice were treated at P0 with MO (80 μg/g) and 0.9% normal saline, respectively. All mice were sacrificed at end stage (P9).

### Statistical analysis

Statistical analyses were performed with GraphPad Prism v5.0 (GraphPad Software). Statistical significance was determined using either Mann-Whitney tests or Friedman two-way ANOVA tests. Kaplan-Meier survival curves were compared and assessed for differences using the log-rank test equivalent to the Mantel-Haenszel test. All data were expressed as mean ± SEM.

## Results

### NMJ Denervation in Different Muscles of Taiwanese Severe SMA Mice at End Stage

**Fig 1:**
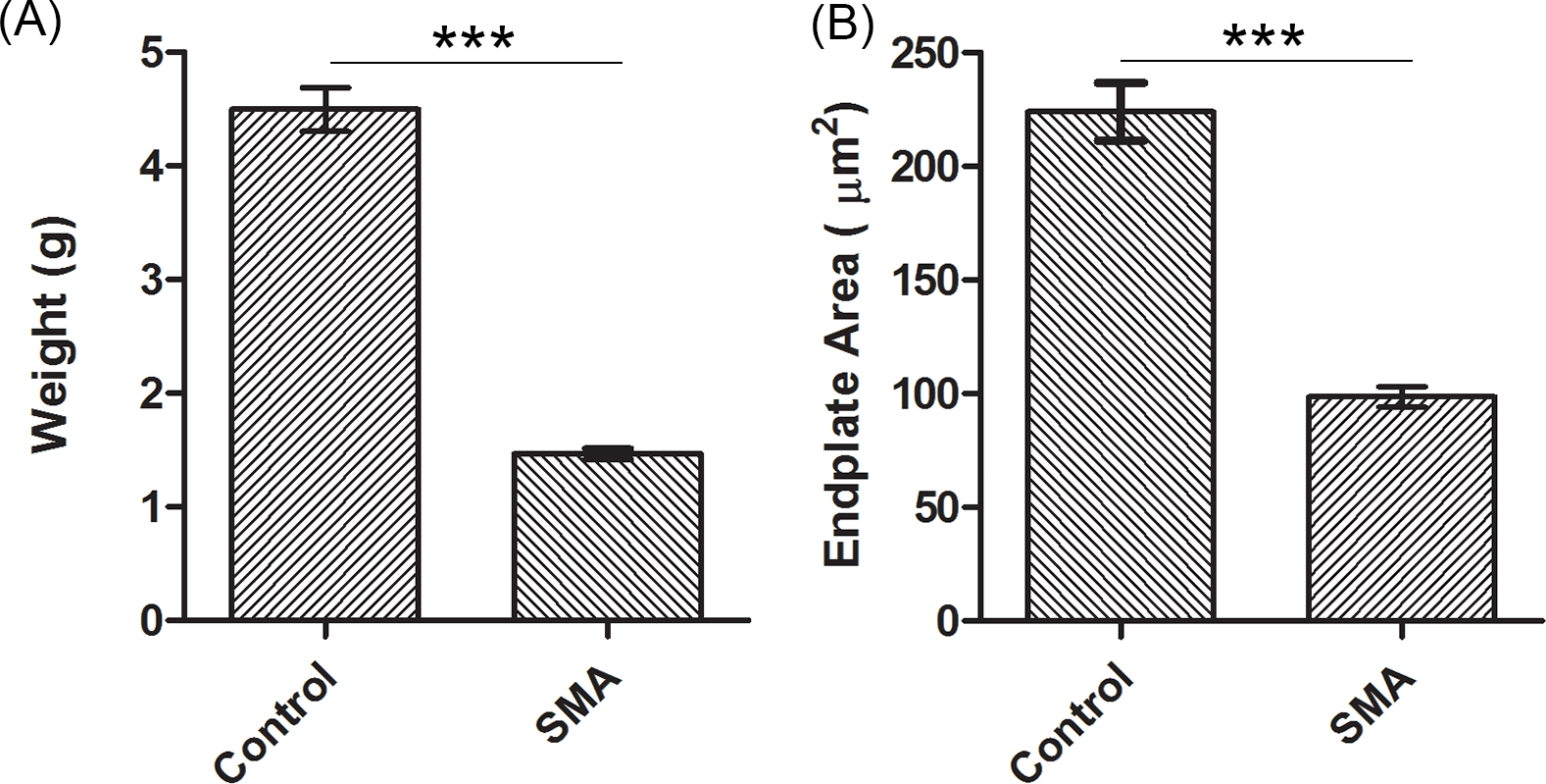
**The differences in (A) weight and (B) endplate area between control and severe SMA mice at P9.** (A) Bar graphs showing the difference in weight between control (n = 34) and severe SMA mice (n = 23) at end stage (P9). (B) Endplate size of the AT muscle in control mice and severe SMA mice at P9. One hundred endplates of the AT muscle were counted in each mouse, and 3 pairs of severe SMA and control mice were quantified with Image J software. All quantitative data are mean ± SEM. ***P < 0.001 versus control mice.

To establish which muscles were highly vulnerable to denervation in Taiwanese severe SMA mice, we examined NMJ denervation patterns in 25 different axial and appendicular muscles. We labelled presynaptic nerve terminals with anti-synaptophysin and anti-neurofilament antibodies and stained postsynaptic endplates with α-bungarotoxin. Control and severe SMA mice were easily identified by phenotype at P9. Compared with control mice, severe SMA mice had a lower weight (P < 0.001) (Fig 1A), and smaller endplate areas (Fig 1B).

**Fig 2:**
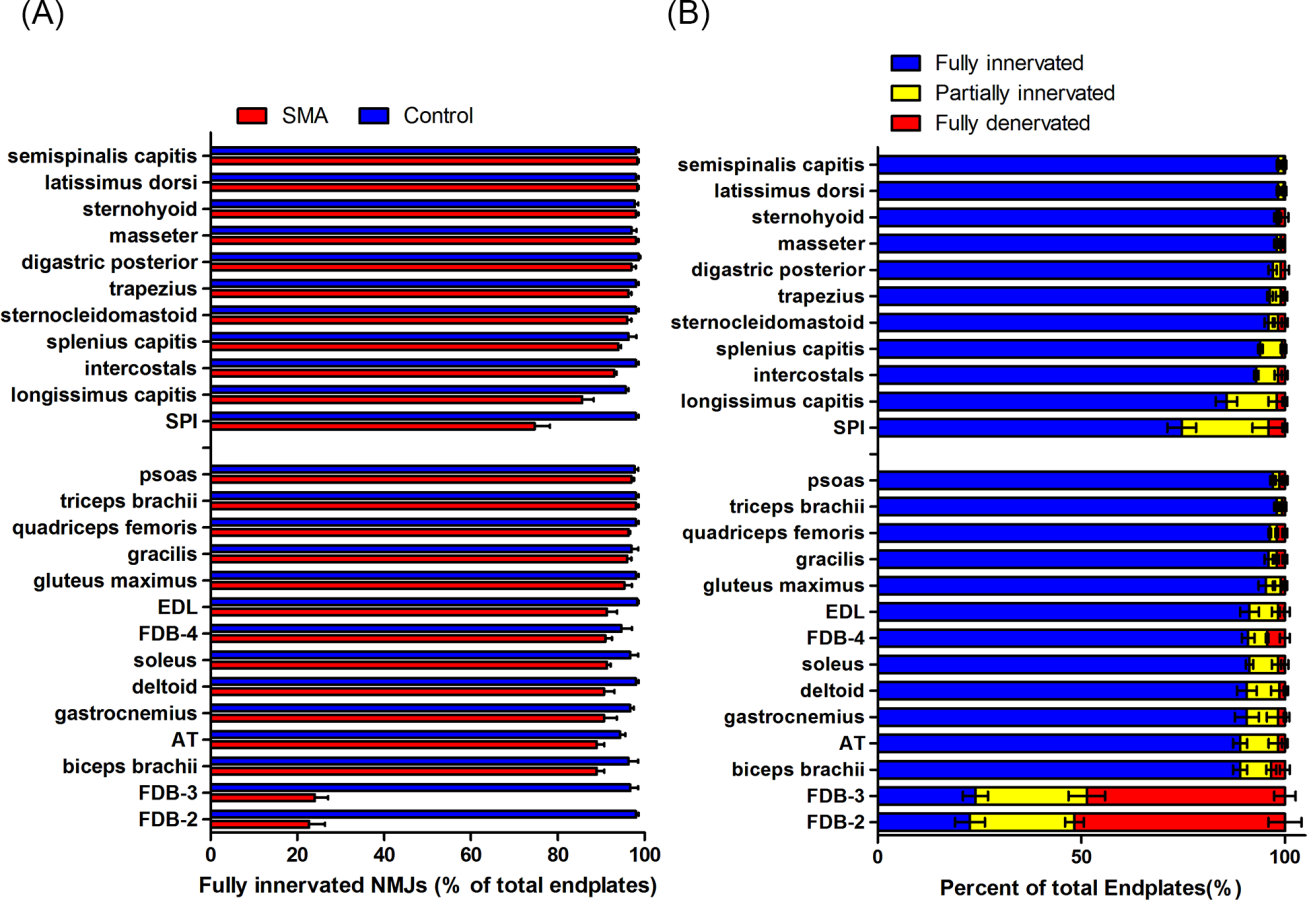
NMJ denervation in different pattern muscles of severe SMA mice at P9. (A) Quantification of fully innervated endplates in muscles of control (blue) and severe SMA (red) mice at end stage (P9). (B) Quantification of fully innervated endplates (blue), partially denervated endplates (yellow), and fully denervated endplates (red) in muscles of severe SMA mice at P9. One hundred NMJs of individual muscles were counted in each mouse, 3 pairs of severe SMA and control mice were studied. All quantitative data are mean ± SEM. (FDB, flexor digitorum brevis; AT, anterior tibialis; EDL, extensor digitorum longus; SPI, serratus posterior inferior)

Mature NMJs in control mice were all fully innervated, as shown by overlapping presynaptic nerve terminals and AChRs. The neurofilament signals were innervated into the endplates, and synaptophysin was evenly distributed and completely covered the endplates. In contrast, partially denervated endplates were only partially covered by presynaptic nerve terminal signalling. Fully denervated endplates were not stained with any presynaptic labelling. Compared with control mice, severe SMA mice showed severe NMJ denervation (< 50% fully innervated endplates), which occurred selectively in appendicular FDB-2 and FDB-3 (FDB-2/3) muscles at P9 (Fig 2A). The percentages of fully innervated, partially innervated, and fully denervated endplates in muscles of severe SMA mice compared to control mice are shown in Fig 2B. The FDB-2/3 muscles displayed severe denervation, averaging 51.7% ± 4.1% and 48.7% ± 2.6% of endplates, respectively. Interestingly, FDB-4 showed slight denervation, averaging 4.3% ± 1.2% of endplates.

**Fig 3:**
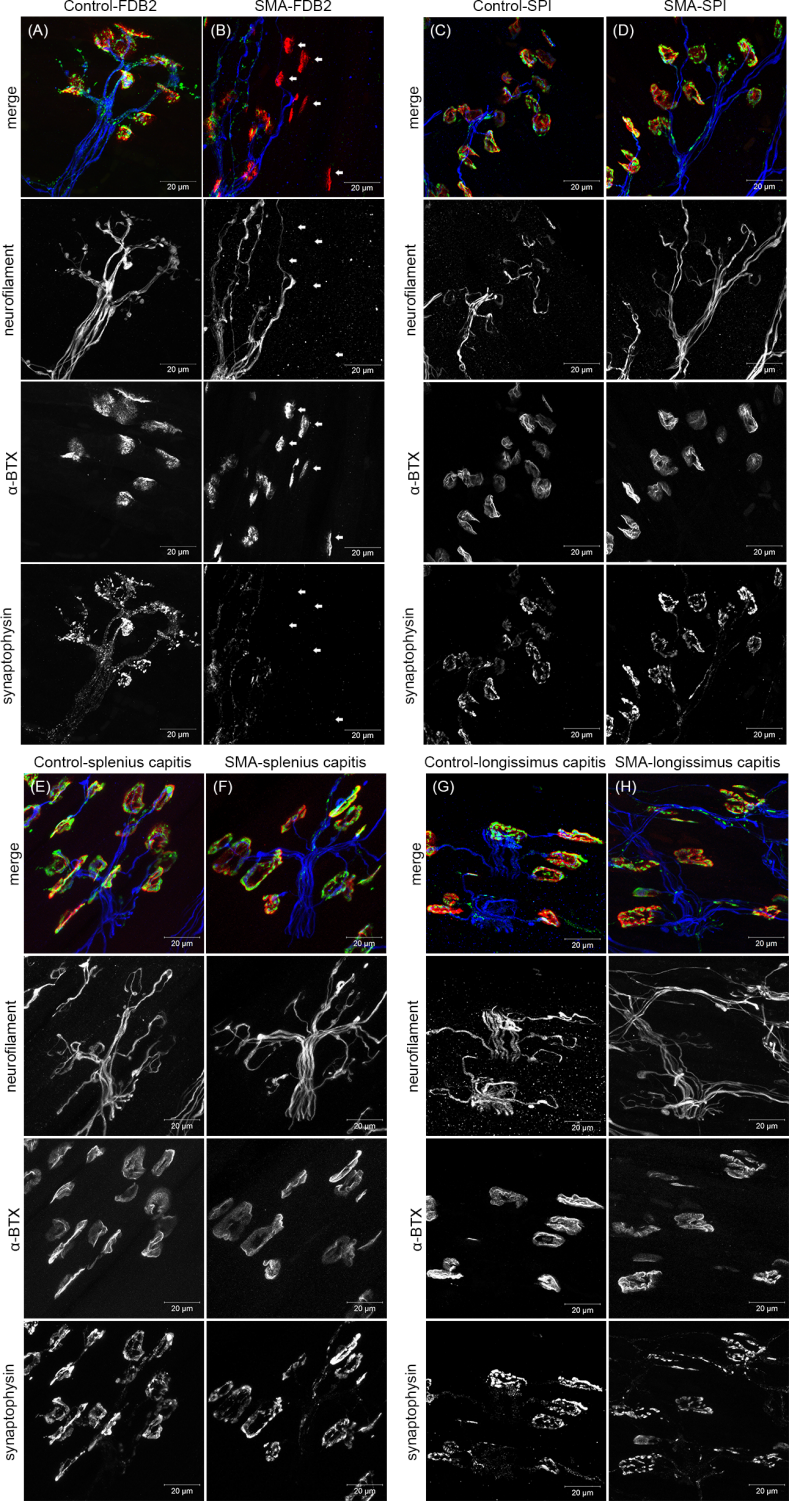
The full denervation of endplates is observed on the FDB-2 muscle of severe SMA mice at P9. The confocal images are Z-stack projection images, stained with anti-synaptophysin and anti-neurofilament antibodies and conjugated α-BTX protein, in control and Taiwanese severe SMA mice at P9. The images in parallel show individual channel grey-scale images and merge images. (A, B) FDB-2 muscle from control (n = 3) and severe SMA mice (n = 3). Most NMJs are fully denervated (arrow) or partially innervated in the FDB-2 muscle of severe SMA mice. (C, D) SPI muscles from control mice and severe SMA mice, (E, F) splenius capitis muscles from control mice and severe SMA mice, and (G, H) longissimus capitis muscles from control mice and severe SMA mice show little difference. (FDB, flexor digitorum brevis; SPI, serratus posterior inferior; α-BTX, α-bungarotoxin).

Compared with control mice, the FDB-2 muscle of severe SMA mice showed a relative deficiency of synaptophysin and neurofilament signals (Fig 3A and 3B). The FDB-3 muscle of severe SMA mouse also showed similar neuromuscular denervation pattern (data not shown). Other muscles, including the SPI, splenius capitis, and longissimus capitis, which have been shown to be severely NMJ denervated in Δ7 mice [15, 17], were not denervated in Taiwanese severe SMA mice in this study (Fig 3C–3H).

### Increased NMJ Denervation of FDB-2/3 muscles of Severe SMA Mice along Disease Progression

**Fig 4:**
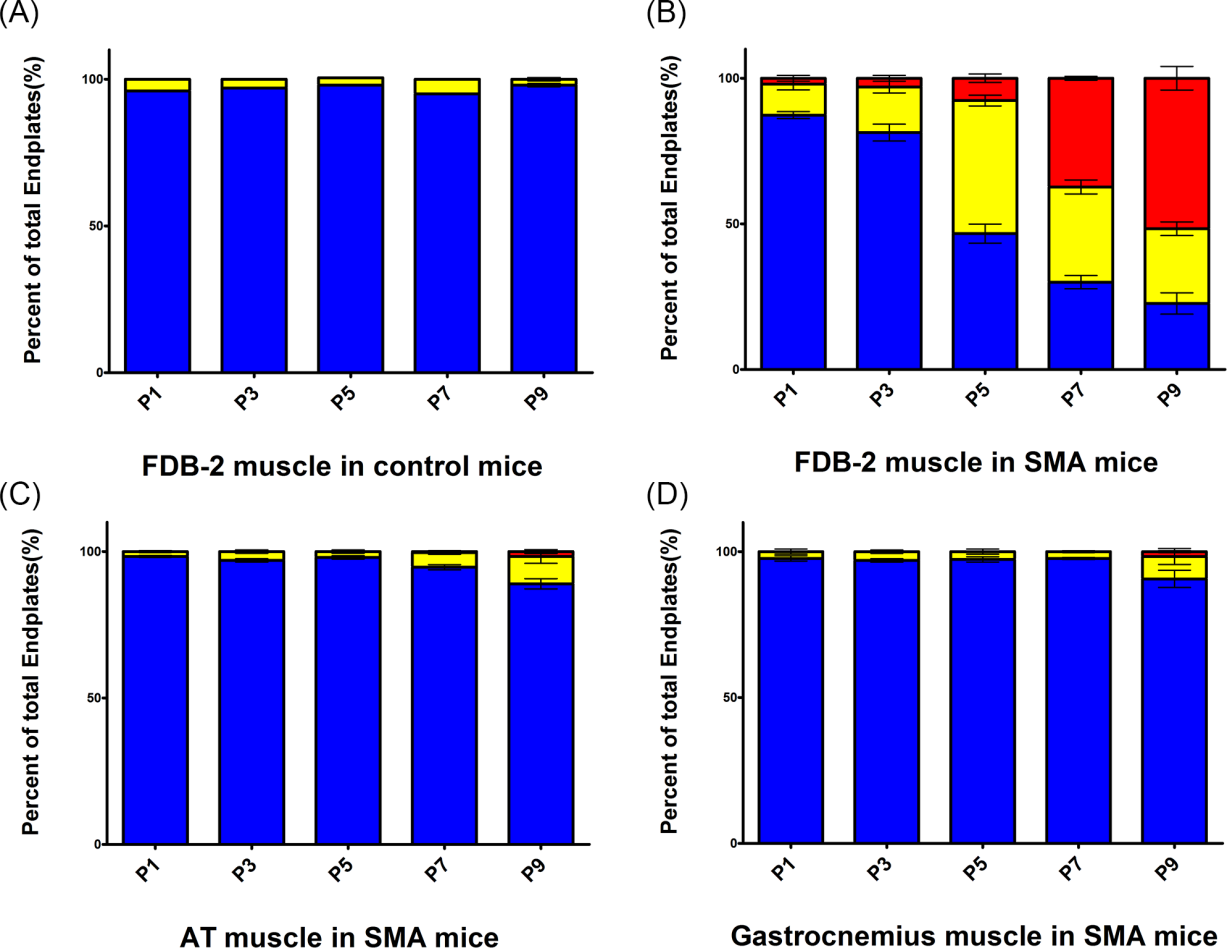
NMJ denervation of FDB-2 muscle is increased with disease progression in Taiwanese severe SMA mice. Bar graphs show fully innervated endplates (blue), partially denervated endplates (yellow), and fully denervated endplates (red) and presented as a percentage of total endplate numbers. (A) FDB-2 muscle in control mice, (B) FDB-2 muscle in severe SMA mice, (C) AT muscle in severe SMA mice, and (D) gastrocnemius muscle in severe SMA mice from P1 to P9. One hundred NMJs of individual muscles were counted in each mouse, and 3 pairs of severe SMA and control mice were studied in P1, P3, P5, P7 and P9, respectively. All quantitative data are mean ± SEM.

NMJ denervation in the severe SMA mouse model has been shown to occur as a failure of synaptic maintenance [12, 17, 35]. To characterize whether denervation in vulnerable muscles of Taiwanese severe SMA mice was also caused by the failure of synaptic maintenance, we planned a time course experiment and collected muscles from P1 to P9 in both control and Taiwanese severe SMA mice. The FDB-2 muscle of control mice did not show significant denervation from P1 to P9 (Fig 4A). In contrast, neuromuscular denervation in the FDB-2 muscle of Taiwanese severe SMA mice increased in severity during disease progression (Fig 4B). The percentages of denervation in FDB-2 muscle of Taiwanese severe SMA mice in P1, P3, P5, P7, and P9 were 2.0 ± 1.0%, 3.0 ± 1.0%, 7.7 ± 1.5%, 37.3 ± 0.7%, and 51.7% ± 4.7%, respectively. The FDB-3 muscle of SMA severe mice showed a similarly increased neuromuscular denervation pattern from P1 to P9 (data not shown). Significant denervation from P1 to P9 was not observed in the AT and gastrocnemius muscles of Taiwanese severe SMA mice (Fig 4C and 4D).

### Neuromuscular Denervation of FDB-2/3 Muscles of Severe SMA Mice is Ameliorated by MO Antisense Oligonucleotide Treatment

**Fig 5:**
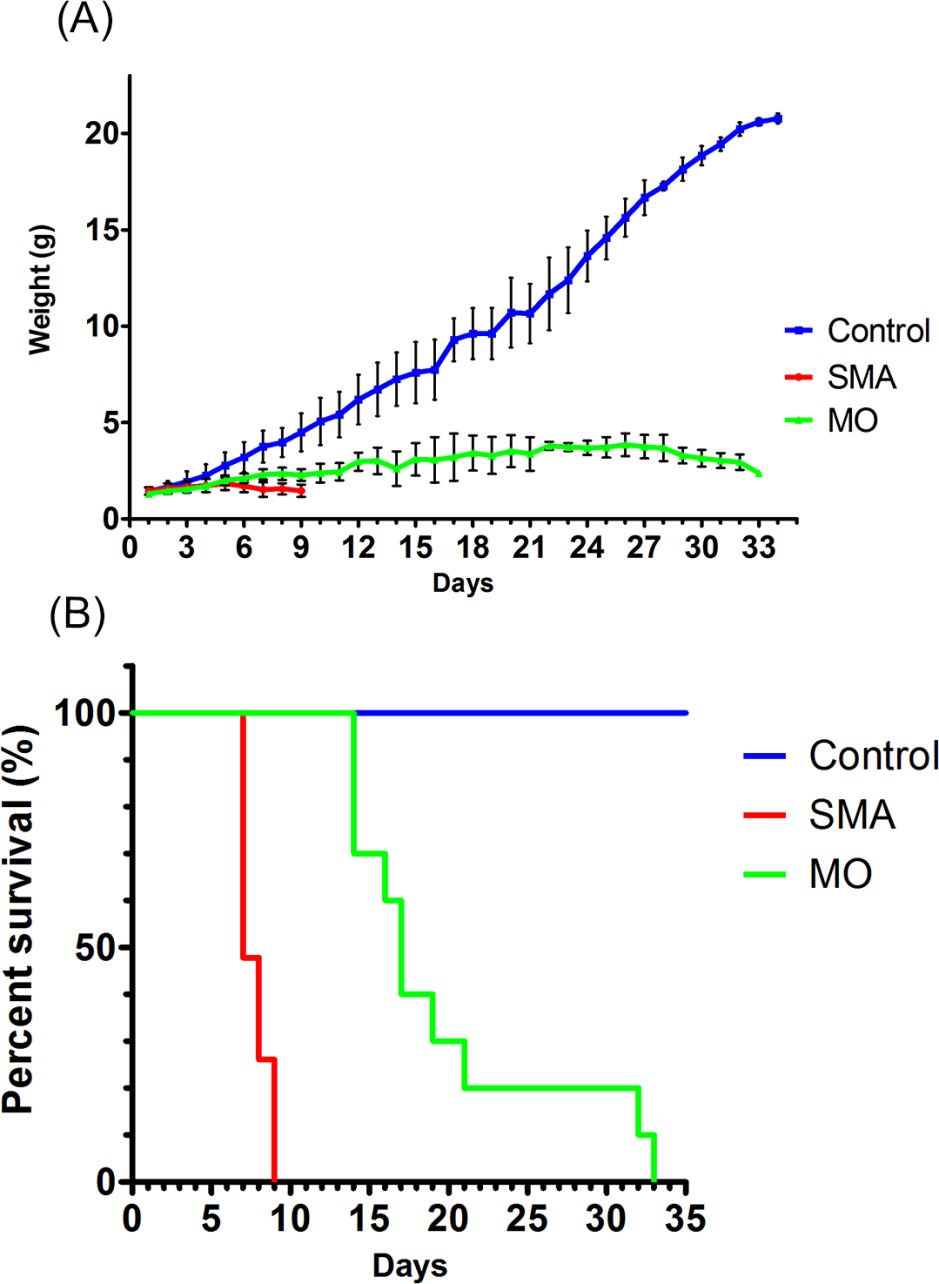
MO treatment increases weight and survival rate in severe SMA mice. (A) Control mice gained weight as expected and MO treatment increased weight compared with untreated SMA mice. Control (blue; n = 34), Taiwanese severe SMA mice (red; n = 23), and Taiwanese severe SMA mice with MO treatment (green; n = 10). All quantitative data are presented as mean ± SEM. (B) Kaplan-Meier curves indicate a dramatic improvement in survival after treatment with MO in Taiwanese SMA severe mice. *P* < 0.05, log-rank test.

If the denervation of FDB-2/3 muscles can be recovered by effective treatment, evaluation of NMJ denervation severity in severe SMA mice may provide a drug-screening platform. The MO antisense oligomer acts against intronic splicing silencer N1 (ISS-N1) and alters SMN2 transcript splicing by regulating incorporation of *SMN2* exon 7. Early treatment with MO increases SMN level and restores motor phenotypes [34]. To investigate whether the denervation in FDB-2/3 muscles could be improved by MO treatment, we treated Taiwanese severe SMA mice with MO by SC injection at 80 μg/g body weight. We performed SC injection with MO in severe SMA and control mice after identification by genotyping at P0. After treatment, we examined the NMJ innervation pattern of FDB-2/3 muscles in each experimental group at P9. We found that treatment with MO by SC injection increased the weight (Fig 5A) and extended the lifespan of severe SMA mice (mean lifespan of Taiwanese severe SMA mice with MO treatment: 19.7 days, n = 10; Taiwanese severe SMA mice without any treatment: 7.7 days, n = 23; control: all survivals, n = 34, P < 0.05) (Fig 5B).

**Fig 6:**
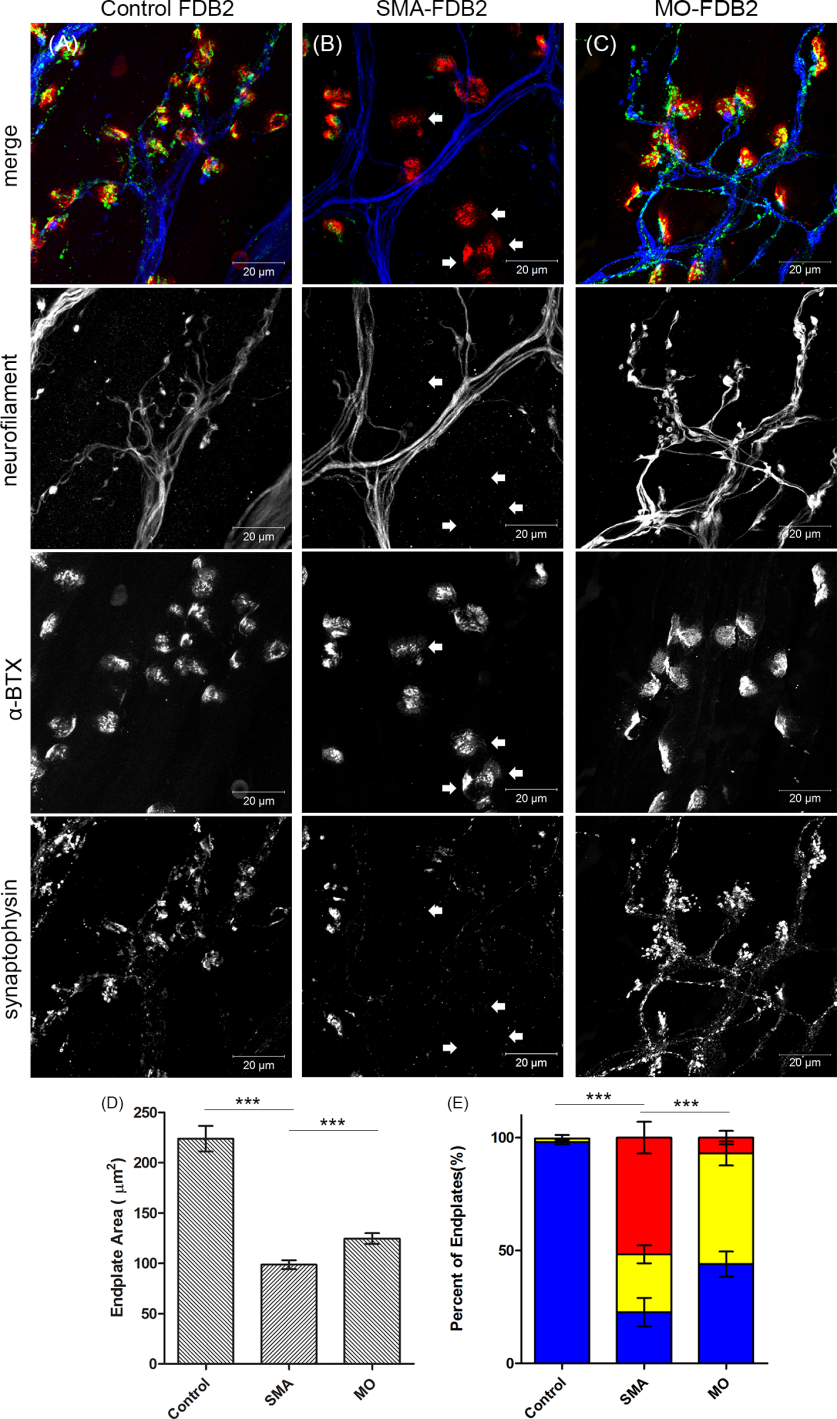
Severe denervation of FDB-2muscle in severe SMA mouse is restored by MO treatment. (A-C) The confocal images are Z-stack projection images at P9, stained with anti-synaptophysin antibody, anti-neurofilament antibody, and conjugated α-bungarotoxin protein from FDB-2 muscles of control, severe SMA, and MO-treated severe SMA mice. The images in parallel show individual channels of grey-scale images and merged images. (A) FDB-2 muscle from control mice (n = 3). NMJs are partially or fully innervated. (B) FDB-2 muscle from severe SMA mice (n = 3). Most NMJs are fully denervated (arrow). (C) FDB-2 muscle from severe SMA mice with MO treatment. Recovery is observed after MO treatment by SC injection at P0. (D) The endplate size of AT muscle in severe SMA mice, MO-treated SMA mice, and control mice at P9. (E) Quantification of fully innervated endplates (blue), partially denervated endplates (yellow), and fully denervated endplates (red) in FDB-2 muscles of control, severe SMA, and MO-treated severe SMA mice at P9. One hundred of individual muscles were counted in each mouse, and 3 pairs of control, severe SMA and MO-treated severe SMA mice were studied. All quantitative data are mean ± SEM, ***P < 0.001.

We also found that the denervation of FDB-2 muscle could be reversed by MO treatment. Compared to the controls, MO treatment increased the synaptophysin signal (Fig 6A–6C). The synaptophysin signal of MO-treated SMA mice was present at nerve terminals, but in clusters, unlike the control group. MO treatment also increased weight (Fig 5A) and endplate size (P < 0.001) in severe SMA mice (Fig 6D). The FDB-2 muscle of severe SMA mice treated with MO had an average of 7.0% NMJ denervation at P9, and an average 44.7% restoration of NMJ denervation compared to untreated Taiwanese SMA severe mice (Fig 6E).

## Discussion

Our study yielded several findings that extend the understanding of the NMJ pathology of Taiwanese severe SMA mice. We observed not only severe denervation, but also increased severity of denervated NMJs from P1 to P9 in the FDB-2/3 muscles of Taiwanese severe SMA mice. These important results suggest that severe NMJ denervation occurs selectively in vulnerable appendicular FDB-2/3 muscles and is likely to start from distal limb muscles in Taiwanese severe SMA mice. Our morphological analysis suggests that SMA may cause a defect in synapse maintenance and denervation that correlates with disease progression. Moreover, we observed neurofilament accumulation at nerve terminals in the FDB-2/3 muscles of Taiwanese severe SMA mice. Finally, early treatment of severe SMA mice with MO by P0 reversed NMJ denervation in FDB-2/3 muscles. Taking these findings together, the denervation which occurs selectively in clinically relevant muscles, like the FDB-2/3 muscles, can provide a suitable platform to uncover the mechanisms of NMJ loss and serve for drug screening in the SMA preclinical stage.

### Severe and Selective NMJ Denervation in Taiwanese Severe SMA Mice

Neurofilament accumulation at the presynaptic terminal is considered to be a critical pathological marker of NMJs in SMA mice and human patients [8–12, 36, 37]. Recent studies also indicated synaptic abnormalities in several hindlimb muscles, such as deficiency in transmitter release and significant loss of synaptic inputs in the motor neurons in Δ7 mice [15, 38]. The decrease of neurotransmitter release was significantly observed in motor nerve terminals of another mild homozygous A2G SMA mouse [14]. Several muscles, including the AT, EDL, and soleus muscles, remained innervated even as SMA progressed [9, 16]. However, other muscles, such as the FDB, SPI, splenius capitis, and longissimus capitis, have exhibited significant NMJ loss at the end stage in Δ7 mice [15, 17]. In the Taiwanese SMA mouse, neuromuscular transmission failures and smaller endplate sizes were shown in mild mice [23], and neurofilament accumulation was evident in severe and intermediate mice [24, 25]. Our study provides additional evidence to characterize the motor neuron innervation in the NMJs of these mice. We systematically examined the NMJs in Taiwanese severe SMA mice and revealed severe NMJ denervation occurring exclusively at distal hindlimb FDB-2/3 muscles. However, we did not observe any significant severe denervation in other clinically relevant muscles examined, including the masseter, SPI, and longissimus capitis, which previously showed severe NMJ denervation in Δ7 SMA mice. The muscles we examined were mostly innervated, including the splenius capitis and longissimus capitis, which are responsible for head movement, the masseter and digastric posterior, which are involved in mastication and swallowing, and the SPI and intercostals, which are involved in respiratory function. These findings indicated that other factors are involved in the premature death of Taiwanese severe SMA mice. Necropsy findings of abnormalities in the intestine, heart, lung, and skeletal muscle vasculature may explain death in Taiwanese SMA severe mice [39, 40]. A recent study showed that full-length SMN depletion caused selective vulnerability of motor neurons [41]. Severe neuromuscular denervation of the FDB-2/3 muscles may contribute to motor deficits and defects in motor neuron pools [15, 38]. Several studies have also suggested that SMA and ALS may share some common biochemical pathways [42–44]; the NMJ is a crucial therapeutic target in both SMA and ALS. Both diseases have different patterns of selective vulnerability determining the denervation of NMJs [17, 45–47]. In ALS, larger motor units innervating fast-twitch muscles denervate first [18, 45]. However, our results suggested the denervation of Taiwanese severe SMA mice started from the terminals of distal appendicular muscles. Furthermore, these experimental models may be specifically beneficial in locating the different factors that determine the susceptibility of NMJs to denervation in SMA.

It is still unclear why selective denervation occurred in such distal and peripheral muscles as FDB-2/3 in the Taiwanese severe SMA mice. Indeed, mouse models of SMA demonstrate additional pathological features that are rarely reported in patients with severe forms of SMA. In Taiwanese SMA mice, intermediate and mild SMA mice show chronic necrosis starting from the tip of the tail and moving toward the root [7]. This necrosis can be treated by SC injection with ASOs [48, 49]. Similarly, after trichostatin A treatment, survival-prolonged Δ7 mice also showed progressive vascular necrosis [50]. These findings suggest that vascular dysfunction is likely a consequence of SMN deficiency. It is noteworthy that distal necrosis with vascular dysfunction has recently been observed in some patients with SMA type I [51–53]. Peripheral muscle or vascular involvement might thus be a unique and early finding in SMA animal models. Further work is required to clarify the function of SMN in vessels and determine how vascular dysfunction may contribute to NMJ dysfunction in SMA.

Collectively, our results show that the majority of axial muscles were still innervated and the distal FDB-2/3 muscles were significantly denervated. These results suggest that the NMJs denervation may start in the distal FDB-2/3 muscles. In addition, SMN deficiency is likely to cause failure of transport or maintenance at nerve terminals.

### Early Treatment and Preclinical Drug Screening

Preclinical screening for SMA treatments depends not only on the rescue of motor neuron loss, but also on the efficiency of restoring NMJ morphology and function. A number of studies have used Taiwanese SMA model mice for clinical testing, including the use of ASOs. For example, agents aimed at augmentation of *SMN2* expression, such as histone deacetylase (HDAC) inhibitor treatment, have been demonstrated to ameliorate motor deficits and increase lifespan in Taiwanese SMA model mice [28, 54, 55]. In the previous study, MO-ASO (10–29) treatment was shown to increase full-length SMN expression, weight gain, and survival time in SMA mice [34]. We observed that the severity of NMJ denervation rapidly increased as the mice aged. After MO administration at P0, NMJ denervation in FDB-2/3 muscles was effectively improved. Based on these findings, severely denervated FDB-2/3 muscles may represent an ideal target for in vivo drug screening and evaluation of therapeutic efficacy in Taiwanese severe SMA model mice.

We concluded that NMJ denervation occurred in the early stage in Taiwanese severe SMA model mice and could be reversed by MO treatment. Restoration of the SMN protein can repair the denervation of NMJs, particularly in FDB-2/3 muscles, which may provide a suitable platform for drug screening in SMA.
